# Temporary conductive hearing loss in early life impairs spatial memory of rats in adulthood

**DOI:** 10.1002/brb3.1004

**Published:** 2018-05-31

**Authors:** Han Zhao, Li Wang, Liang Chen, Jinsheng Zhang, Wei Sun, Richard J. Salvi, Yi‐Na Huang, Ming Wang, Lin Chen

**Affiliations:** ^1^ Hefei National Laboratory for Physical Sciences at the Microscale School of Life Sciences University of Science and Technology of China Hefei China; ^2^ Auditory Research Laboratory University of Science and Technology of China Hefei China; ^3^ Department of Otolaryngology‐Head and Neck Surgery Wayne State University School of Medicine Detroit Michigan; ^4^ Center for Hearing and Deafness State University of New York at Buffalo Buffalo New York

**Keywords:** neurogenesis, NMDA receptor, spatial memory, synaptic plasticity, temporary conductive hearing loss

## Abstract

**Introduction:**

It is known that an interruption of acoustic input in early life will result in abnormal development of the auditory system. Here, we further show that this negative impact actually spans beyond the auditory system to the hippocampus, a system critical for spatial memory.

**Methods:**

We induced a temporary conductive hearing loss (TCHL) in P14 rats by perforating the eardrum and allowing it to heal. The Morris water maze and Y‐maze tests were deployed to evaluate spatial memory of the rats. Electrophysiological recordings and anatomical analysis were made to evaluate functional and structural changes in the hippocampus following TCHL.

**Results:**

The rats with the TCHL had nearly normal hearing at P42, but had a decreased performance with the Morris water maze and Y‐maze tests compared with the control group. A functional deficit in the hippocampus of the rats with the TCHL was found as revealed by the depressed long‐term potentiation and the reduced NMDA receptor‐mediated postsynaptic current. A structural deficit in the hippocampus of those animals was also found as revealed the abnormal expression of the NMDA receptors, the decreased number of dendritic spines, the reduced postsynaptic density and the reduced level of neurogenesis.

**Conclusions:**

Our study demonstrates that even temporary auditory sensory deprivation in early life of rats results in abnormal development of the hippocampus and consequently impairs spatial memory in adulthood.

## 1 INTRODUCTION

The brain development is strongly shaped by sensory experience (Rittenhouse, Shouval, Paradiso, & Bear, 1999; Rosenzweig & Bennett, 1996), including the auditory sensory experience, particularly at the early age (Gao & Suga, 2000; Kilgard et al., 2001). In a condition of deprived hearing, the development of the central auditory system is substantially affected. Auditory sensory deprivation during development resulting from a temporary and reversible unilateral conductive hearing loss can distort tonotopic maps, weaken the deprived ear’s representation, strengthen the open ear’s representation, and disrupt binaural integration of interaural level differences (Polley, Thompson, & Guo, 2013; Popescu & Polley, 2010). Conductive hearing loss can disrupt synaptic and spike adaptation in the developing auditory cortex (Xu, Kotak, & Sanes, 2007). Auditory deprivation resulting from cochlear removal at the early age leads to structural changes in the central auditory systems, such as reduced volume of cochlear nucleus and neuronal apoptosis in the cochlear nucleus (Mostafapour, Cochran, Del Puerto, & Rubel, 2000).

Most studies focus on effects of auditory deprivation on the auditory systems; however, accumulating evidence suggests that these effects may span beyond the auditory system. Deaf children show alterations in fine motor skills (Horn, Fagan, Dillon, Pisoni, & Miyamoto, 2007; Horn, Pisoni, & Miyamoto, 2006). They pay more attention to the peripheral visual field (Bavelier et al., 2000; Bottari, Nava, Ley, & Pavani, 2010; Proksch & Bavelier, 2002), which leads to deficits in sustained attention (Barker et al., 2009; Yucel & Derim, 2008). It remains unclear whether or not there is a causal relationship between auditory deprivation and the nonauditory deficits. In this study, we evaluated consequences of hearing deprivation resulting from temporary conductive hearing loss in early life (postnatal 14) on spatial memory of rats in adulthood. In addition, the mechanism underlying the impaired spatial memory in these rats was further explored by the functional and structural assessment of the hippocampus.

## 2 MATERIALS AND METHODS

### 2.1 Animal models

We collected data from a total number of 203 male Wistar rats. We purchased pregnant female rats from Vital River Laboratories, Beijing, China, and their male offspring were used in our study. We randomly classified the offspring into the temporary conductive hearing loss (TCHL) group and the control group (Table 1). The eardrums of the rats of the TCHL group were perforated bilaterally at 14 postnatal days to develop early age conductive hearing loss. The method of eardrum perforation surgery was similar to that described previously (Sun et al., 2011). In brief, the surgery was conducted under an upright surgical microscope, and the rats were under slight anesthesia with ethyl ether. The eardrum was visualized under the microscope. The entire eardrum was ruptured, without damaging the ossicular chain, cochlea, and/or other structures. The rats of the control group underwent a sham surgery during which they were anesthetized with ethyl ether in the absence of eardrum perforation. All animals were then returned to their cages, housed in standard housing conditions at 12/12‐hour light/dark cycles with food and water ad libitum. The use and care of animals in this study followed the protocols approved by the Institutional Animal Care and Use Committee of University of Science and Technology of China (Approval number: USTCACUC1402021).

**Table 1:** Number of animal subjects allocated for each experiment

Experiment	Number of subjects	
	Control	TCHL
ABR	8	8
Open‐field test and Morris water maze test	14	15
Y‐maze test	10	10
Field potential recording	19	19
Patch‐clamp recording	20	19
Western blot	9	9
BrdU immunohistochemistry	6	6
Golgi‐Cox staining	6	6
Transmission electron microscopy	8	10
CTB retrograde tracing	1	
	Total: 203	

ABR: auditory brainstem response; CTB: cholera toxin subunit B; TCHL: temporary conductive hearing loss.

### 2.2 Auditory brainstem response (ABR)

ABR testing was performed to evaluate hearing thresholds of rats with a TDT electrophysiology platform (Tucker‐Davis Technologies, RRID:http://scicrunch.org/resolver/SCR_006495, USA). The rats were placed on a soft pad in a soundproof chamber after being anesthetized with 8% chloral hydrate (4 ml/kg, administered intraperitoneally). Although chloral hydrate was used in this study, we are now aware that the use of this drug as an anesthetic is discouraged (Baxter, Murphy, Taylor, & Wolfensohn, 2009) and we will not use it in our future studies. Acoustic stimuli (clicks) generated with TDT System 3 (RP 2.1, PA 5, ED 1) decreased from 80 dB SPL at 5 dB intervals. The acoustic stimuli were presented in a closed field with a TDT electrostatic speaker ES 1. A total of three subcutaneous stainless steel needle electrodes were positioned at the vertex (positive), contralateral mastoid (negative), and nose tip (ground) of the animal. The resistance between each electrode and the ground electrode was less than 1 kΩ. The ABRs to the click were recorded with TDT RA16 and stored electronically for off‐line analysis with TDT BioSigRZ.

### 2.3 Behavioral tests

Behavioral tests were carried out in a soundproof room. Each test was conducted during the light phase of the light/dark cycle in the following order: open‐field test, Y‐maze test, and Morris water maze test. All behavioral tests were recorded by a digital camera interfaced to a computer. The data were recorded using the Noldus EthoVision (Noldus, RRID:http://scicrunch.org/resolver/SCR_004074, Wageningen, the Netherlands) video imaging software. Later, videos were analyzed by the Noldus EthoVision.

#### 2.3.1 Open‐field test

The open‐field test was used to detect motor and exploratory behavior of the rats. The open field comprised black wood and a floor (100 cm × 100 cm) with 50‐cm walls. The box floor was painted with white lines in order to form nine equal squares. During a 5‐min observation period, rats were placed in one corner of the apparatus facing the wall. The number of total squares crossed and the rearing frequency were recorded.

#### 2.3.2 Y‐maze test

The Y‐maze test was used to assess short‐term spatial memory and exploratory activity in a novel environment (Soares et al., 2013). It was a three‐arm maze with equal angles among all arms (45 cm × 15 cm × 30 cm). The arms were labeled as follows: the start arm, the other arm, and the novel arm. The experiment consisted of two sessions (training and test) conducted on two successive days. In the training session, the novel arm was closed, and the rats were placed at the end of the start arm facing the wall. The rats were then allowed to freely explore the maze for 10 min. The test session was conducted on the second day. The novel arm was opened, and the rats were allowed to travel freely in all three arms for 5 min. The total distance covered by the rats, the total number of entries, and the time spent in each arm were calculated. The ratio of time/entries in the novel arm to the total arm was calculated by the following formula: [Novel/(Novel + Other) × 100].

#### 2.3.3 Morris water maze test

The Morris water maze test was used to evaluate spatial learning and memory. The maze consisted of a black circular pool (diameter 160 cm, height 50 cm, filled with water at 21–22°C to a height of 30 cm). A black circular platform (9 cm in diameter) was placed 2–3 cm below the water line in the center of one quadrant and remained in the same position. Several constant large visual cues surrounded the tank at a height of 120–150 cm in order to facilitate orientation of the animals. The task consisted of a 5‐day acquisition phase with four massed trials administered each day, and a 1‐day memory retention test phase. During acquisition phase, the rats were placed in the water facing the wall at random start locations, namely north, south, east, and/or west. The locations were at equal distances from each other on the pool rim. Each rat was allowed to find a submerged platform within 90 s and to rest on it for 20 s. If the rat failed to find the hidden platform within 90 s, it was guided to the platform and allowed to remain there for 20 s. The procedure was repeated for all four start locations. On the 6th day (the probe test phase), following the 5 days of acquisition phase, memory retention was determined in a single 90‐s probe trial. The underwater platform was removed. The rats were placed to the water from the opposite quadrant of the platform, facing the wall, and were permitted to explore the environment for 90 s ad libitum. The performance parameters for each rat included the following: total swim distance, mean swim velocity, and the duration in each quadrant. The parameters were recorded and analyzed.

### 2.4 In‐vivo field potential recordings

Electric stimulus‐evoked field potentials were recorded from the CA1 area of the hippocampus in an in‐vivo condition. Rats were anesthetized with urethane (1.5 g/kg), and their heads were fixed in a stereotaxic head holder. The body temperature of the animal was continuously monitored. The skull was subsequently exposed. A concentric bipolar stimulating electrode was placed on the Schaffer fibers (4.2 mm posterior to bregma and 3.8 mm lateral to the midline), and a recording electrode, filled with 2 M NaCl solution, was placed in the stratum radiatum in the CA1 area (3.4 mm posterior to bregma and 2.5 mm lateral to the midline). The depths of the stimulating electrode and recording electrode were adjusted based on the magnitude of the fEPSP response. The EPSP slope of input/output curve (I/O), paired‐pulse ratio (PPR), and long‐term potentiation (LTP) were recorded. The intensity of test stimuli was adjusted to evoke approximately 50% of the maximum response of fEPSP slope in the CA1 area. The fEPSP slope was considered as the maximal slope obtained on the first deflection (negative for CA1) of the potential. The I/O curves were generated by systematic variation of the stimulus current (0.1–1.5 mA) in order to evaluate synaptic potency. The pulses of the stimuli were delivered at 0.05 Hz, and three responses at each current level were used in order to calculate the mean response. PPR was evaluated by systematic variation of the interpulse intervals (IPI, from 10 ms to 800 ms). The pairs of stimuli were delivered at 0.05 Hz, and three responses were used in order to calculate the mean response at each IPI. The fEPSP2/fEPSP1 was defined as the PPR for the quantification of the enhancement and/or inhibition effect of the second response relative to the first. The peak facilitation (the values of the extreme point of the curve) was used to describe the differences in the two groups. Baseline recording of LTP was obtained for 20 min at 0.05 Hz, followed by application of the high‐frequency stimulation (HFS: 5 trains of 20 pulses at 200 Hz separated by 1 s, repeated six times at interval of 1 min). Posttetanic recordings were conducted for 1 hr with single‐pulse stimuli at 0.05 Hz. The responses recorded every 5 min were averaged and were normalized according to baseline values. All data were recorded by the Igor program.

### 2.5 Hippocampal slice preparation

All chemicals used in the following experiments were purchased from Sigma‐Aldrich (USA) unless otherwise specified. Rats were anesthetized with 8% chloral hydrate (4 ml/kg, i.p.), and their brains were dissected. Coronal brain slices (300 μm) were cut with a vibratome (Leica VT‐1200S, Germany) in ice‐cold (0–2°C) N‐methyl‐D‐glucamine artificial cerebrospinal fluid (NMDG aCSF) containing (in mM): 93 NMDG; 93 HCl; 2.5 KCl; 1.2 NaH_2_PO_4_; 30 NaHCO_3_; 25 Glucose; 20 HEPES; 5 Na‐ascorbate; 3 Na‐pyruvate; 2 Thiourea; 10 MgSO_4_; 0.5 CaCl_2_.2H_2_O; and 3 GSH. Later, the slices were transferred to a recovery chamber, which contained NMDG aCSF equilibrated with 95% O_2_/5% CO_2_ for 10–12 min at 32–34°C. After that, the slices were transferred to a holding chamber with HEPES aCSF for approximately 1 hr at room temperature (26–28°C) until further recording. HEPES aCSF (in mM): 92 NaCl; 2.5 KCl; 1.2 NaH_2_PO_4_; 30 NaHCO_3_; 25 Glucose; 20 HEPES; 5 Na‐ascorbate; 3 Na‐pyruvate; 2 Thiourea; 10 MgSO_4_; 0.5 CaCl_2_.2H_2_O; and 3 GSH. The slices were transferred to a recording chamber under continuous perfusion (~2 mL/min) with oxygenated standard aCSF (in mM): 129 NaCl; 3 KCl; 1.2 KH_2_PO_4_; 1.3 MgSO_4_; 20 NaHCO_3_; 2.4 CaCl_2_; 3 HEPES; and 10 glucose. All solutions were in the range 300–310 mOsm and the pH 7.3–7.4.

#### 2.5.1 Patch‐clamp recordings

Whole‐cell recordings were obtained with patch electrodes that exhibited tip resistance of 3–5 MΩ when filled with an internal solution containing (in mM): 130 Cs‐methSO_3_; 0.15 CaCl_2_; 2.0 MgCl_2_; 2.0 EGTA; 10 HEPES; 2.0 Na_2_‐ATP; 0.25 Na_3_‐GTP; and 10 lidocaine N‐ethyl bromide (QX‐314). The pH of the solution was adjusted to 7.2–7.3 with CsOH, and the osmolarity was 280 mmol/kg with sucrose. The stimulating electrode was placed in the Schaffer collateral (SC), while the recording glass electrode was placed in the CA1 pyramidal neurons. The evoked EPSCs were initially recorded at a holding potential of V_h_ = −80 mV in order to assess AMPA receptor‐mediated responses. The NMDA receptor‐mediated EPSCs were recorded at V_h_ = +40 mV in the presence of CNQX (10 μM), an AMPA receptor antagonist. All EPSCs were recorded in the presence of picrotoxin (100 μM), a GABA_A_ receptor antagonist that is used to suppress GABA_A_‐mediated currents. During the recording for miniature excitatory postsynaptic currents (mEPSCs), the electrodes were filled with the internal recording solution containing (in mM): 130 K‐gluconate, 2 MgCl_2_, 5 KCl, 0.6 EGTA, 10 HEPES, 2 Mg‐ATP, and 0.3 Na‐GTP. The pH of the solution was adjusted to pH 7.3 with additions of KOH, and the osmolarity was adjusted to the range of 285–295 mmol/kg. The tip resistance ranged between 3 and 5 MΩ. Following initial characterization in the current clamp, all experiments were conducted in a voltage clamp at a holding potential of ‐60 mV. CNQX (10 μM, an AMPA receptor antagonist), picrotoxin (100 μM), and tetrodotoxin (TTX, 1 μM, the voltage‐gated sodium channel blocker) were applied to pharmacologically isolated NMDA receptor‐mediated mEPSCs. The data were discarded when the access resistance varied by a percentage higher than 20% (>20%) during the recording.

#### 2.5.2 Western blot

The left and right hippocampi were removed within 15 min following in‐vivo field potential recordings for western blot. The tissue samples were homogenized in 200 μl of buffered isotonic cocktail containing protease and phosphatase inhibitors. The lysis solution contained (in mM): 150 NaCl, 0.075 pepstatin, 0.1 leupeptin, 1 PMSF, 5 benzamidine, 1 EDTA, 1 EGTA, 20 Tris, 15 Na_4_P_2_O_7_, 100 B‐glycerophosphate, and 25 NaF. The homogenates were incubated for 45 min on ice and then centrifuged at 12,500 × *g* for 25 min at 4°C in order to obtain the supernatant proteins. Total protein was estimated in sonicated samples by the BCA assay (Pierce Chemical Rockford, IL, USA), and the samples were diluted with buffer in order to contain the same concentration of protein (5 μg/20 μl). The samples were boiled for 10 min and stored at −20°C until further use. The samples that contained 5 μg of proteins were resolved in the 10% SDS‐acrylamide gels and run at 120 V. The proteins were separated according to their molecular weight. The separated proteins on the gel were transferred to PVDF membranes (Millipore, USA) and incubated with 10% fat‐free milk for 1 hr at room temperature. The blots were initially incubated overnight at 4°C with primary antibodies as follows: anti‐GAPDH (Millipore Cat# AB2302, RRID:http://scicrunch.org/resolver/AB_10615768, USA), anti‐NR2A (Millipore Cat# 05‐901R, RRID:http://scicrunch.org/resolver/AB_11215116, USA), and anti‐NR2B (Millipore Cat# MAB5780, RRID:http://scicrunch.org/resolver/AB_838222, USA). The membranes were extensively washed with Tris‐Buffered Saline Tween‐20 (TBST) three times each for 10 min and incubated for 2 hr at room temperature with a secondary antibody conjugated with horse radish peroxidase (HRP). The secondary antibodies used were the following: anti‐chicken IgY, HRP conjugate (Promega Cat# G1351, RRID:http://scicrunch.org/resolver/AB_430845, USA) and anti‐rabbit IgG, HRP conjugate (Promega Cat# W4011, RRID:http://scicrunch.org/resolver/AB_430833, USA).

#### 2.5.3 BrdU administration and immunohistochemistry

The BrdU assay was used in order to evaluate neurogenesis of the hippocampus. The rats received intraperitoneal injections of 5‐ bromo‐2′‐deoxyuridine (BrdU, 100 mg/kg) (dissolved in a solution containing 0.9% NaCl and 0.007 M of NaOH) on the second day of the eardrum perforation surgery. The animals were sacrificed and perfused through the heart with 0.9% NaCl, fixed with 4% ice‐cold paraformaldehyde (PFA) at 7 days (P21) or 4 weeks (P42) following the eardrum surgery. The brains were subsequently removed, postfixed overnight in the PFA, and equilibrated in 30% sucrose solution. Serial coronal sections of 40 μm were obtained by a freezing stage microtome (Leica CM1950, Germany). The sections were stored in cryoprotectant solution (30% ethylene glycol, 25% glycerol, and 45% of 0.1 M sodium phosphate buffer) at −20°C until further immunohistochemical analysis. For immunoperoxidase staining, free‐floating sections were initially washed thoroughly (5 × 5 min) in 0.1 M of PBS solution (pH 7.4). The sections were incubated for 2 hr in 50% formamide in 2 × SSC (dilute from 20 × SSC to 2 × SSC, SCC: saline sodium citrate) at 65°C in order to achieve denaturation of the proteins (warm water bath). Later, the sections were incubated for 30 min in 3% H_2_O_2_ in PBS in order to block endogenous peroxidase activity and washed three times for 5 min in PBS. The sections were treated with 2 N HCl for DNA denaturation (30 min at 37°C) and rinsed in 0.1 M boric acid for 10 min at room temperature (pH 8.5), rinsed in PBS (3 times for 5 min each), and incubated in blocking solution (3% horse serum and 0.1% Triton X‐100 in PBS,1 hr at room temperature). The sections were then incubated with the primary antibody (monoclonal mouse anti‐BrdU IgG, 1:200, Cat# sc‐32323, RRID:http://scicrunch.org/resolver/AB_626766, Santa Cruz Biotechnology, USA) in blocking solution at 4°C overnight, washed subsequently in PBS 3 times for 5 min, and finally incubated with a biotinylated secondary antibody (horse anti‐mouse IgG, 1:200, Cat# BP‐2000, RRID:http://scicrunch.org/resolver/AB_2687893, Vector Laboratories, Inc., USA) for 2 hr at room temperature. Following incubation with the secondary antibody, the sections were washed three times with PBS for 5 min. The sections were incubated for 2 hr in 1:200 ABC (ABC Elite kit; Vector Laboratories, Inc. USA) diluents at room temperature. The sections were washed once in PBS for 5 min and then visualized using a detection solution (0.05% DAB and 0.003% H_2_O_2_ in 0.05 M Tris‐HCl buffer) for 2–5 min. The sections were rinsed in TBS (6 times for 10 min each) and finally mounted onto gelatine‐coated slides and air‐dried. Every 8th section throughout, the hippocampus was used to determine the total number of BrdU^+^‐labeled cells in the DG (subgranular zone [SGZ] and granule cell layer [GCL]) under light microscopy for each animal. The total number of BrdU^+^ cells counted was multiplied by the section interval of 8 in order to generate the stereological estimate. The resulting cells were counted using Image‐Pro Plus 6.0 software (Image‐Pro Plus, RRID:http://scicrunch.org/resolver/SCR_007369, Media Cybernetics, Inc. USA). BrdU^+^ cell densities were calculated by dividing the total BrdU^+^ cell numbers by the total volume of analysis.

#### 2.5.4 Golgi‐Cox staining

In this study, a modified Golgi‐Cox staining method was used as described in a previous study (Ranjan & Mallick, 2012). The Golgi‐Cox solution contained 5, 5, 4, and 10 volume parts of 5% potassium dichromate solution, 5% mercuric chloride solution, 5% potassium chromate solution, and ddH_2_O, respectively. The rats were sacrificed by cervical dislocation followed by decapitation. All the brains were removed and washed with distilled water followed by freshly prepared Golgi‐Cox solution. The brains were separated into two sections (5 mm thick) via a longitudinal cut along the midline. Each of the coronal blocks was placed in Golgi‐Cox solution at 37 ± 1°C for 48 hr. The brain slices were cut at 100 μm and prepared from brain blocks using a vibratome (Leica VT 1200S, Germany). The brain slices were stained using the following procedures: The samples were rinsed twice (5 min each) in distilled water, dehydrated in 50% alcohol for 5 min, kept in ammonia solution for 10 min, and rinsed for an additional two times (5 min each) in distilled water. The samples were kept in 5% sodium thiosulfate for 10 min in dark, rinsed twice (2 min each) in distilled water, and dehydrated twice (10 min each) in ethanol solutions containing 70%, 80%, and 95% ethanol and in 99% 1‐butanol solution. Later, the samples were cleared in toluene and mounted in dispersed plasticizer xylene on gelatinized slides. At last, the slides were allowed to dry at room temperature and were observed under a microscope (ZEISS Axioskop 2 plus, Germany).

#### 2.5.5 Transmission electron microscopy

Hippocampi were removed following high‐frequency stimulation, fixed in glutaraldehyde for 24 hr at 4°C, and subsequently postfixed in 1% osmium tetroxide for 2 hr at room temperature. The samples were dehydrated, infiltrated, and embedded in conventional epoxy resin. Ultrathin sections were cut and mounted on 200‐mesh copper grids. The sections were stained with uranyl acetate and lead citrate, and then examined for postsynaptic density (PSD) in spines of hippocampal neurons using transmission electron microscopy (TEM) (JEOL‐1230, Tokyo, Japan).

#### 2.5.6 Retrograde tracer injections

The rat was anesthetized with 2% pentobarbital sodium (40 mg/kg) and prepared for stereotaxic surgery. A craniotomy was conducted in the left hippocampus (3.4 mm posterior to the bregma, 2.5 mm lateral from midline, and depth 2.4 mm). Glass micropipettes (15–10 mm tips) filled with 2% cholera toxin subunit B (CTB) conjugated with Alexa Fluor 594 were lowered into place. Under a dissection microscope, a series of injections with CTB (20 nl/min, 50‐100 nl) was conducted to the left hippocampus using a 30‐gauge needle connected to a Hamilton syringe (10 μl) under microscopic guidance. The micropipettes were left in place for 10 min, and the burr hole was packed with sterile bone wax, while the incision was closed with wound clips. The rats were anaesthetized 1 week and then perfused with 100–150 ml 0.9% of NaCl, followed by 250 ml of 4% paraformaldehyde (PFA). Following perfusion, the brain was removed, placed in fixative solution overnight, and subsequently transferred to a 30% sucrose solution for cryoprotection. The serial coronal sections were cut at 40 μm using a freezing stage microtome (Leica CM1950, Germany). The sections were mounted on gelatin‐coated slides. The fluorescent images were captured with a fluorescent microscope (ZEISS Axioskop 2 plus, Germany).

#### 2.5.7 Data analysis

All data were expressed as mean ± *SEM*. The data were analyzed with one‐way or two‐way analysis of variance (ANOVA) followed by Bonferroni’s post‐hoc comparisons test using Origin Pro 8.0 (OriginLab Corporation, RRID:http://scicrunch.org/resolver/SCR_014212, USA). The graphs were drawn using Origin Pro 8.0 and GraphPad Prism software version 5.0 (GraphPad Prism, RRID:http://scicrunch.org/resolver/SCR_002798, San Diego, CA, USA). The differences were considered statistically significant when a *p* value lower than 0.05 was obtained (*p *<* *0.05).

## 3 RESULTS

### 3.1 Perforation of the eardrum induced a TCHL

**Figure 1:**
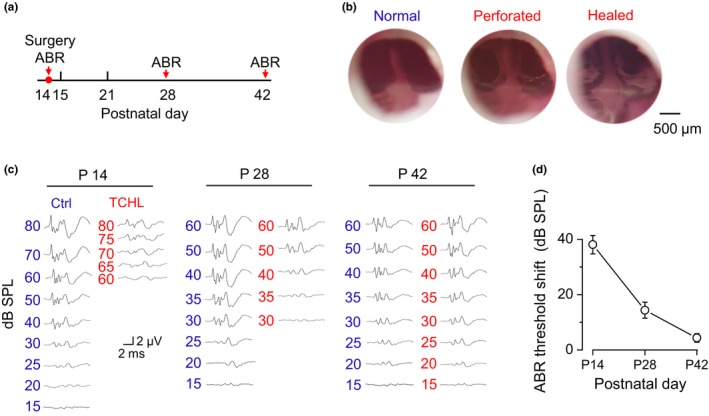
Eardrum perforation induced a TCHL. (a) Time schedule for eardrum perforation surgery and ABR tests. (b) Photographs showing an eardrum before, immediately after (P14), and 28 days after (P42) surgery for eardrum perforation. The perforated eardrum was healed with visible scars in P42 animals. (c) Sample traces of ABR waveforms to acoustic clicks in P14, P28, and P42 rats. (d) Group data of the ABR threshold shifts indicating incomplete recovery of hearing following eardrum perforation (Control: *n* = 8, TCHL: *n* = 8). ABR, auditory brainstem response; TCHL, temporary conductive hearing loss

To induce a TCHL, we surgically perforated the bilateral eardrums of rats at P14 (Figure 1a) and allowed them to heal (Figure 1b). The ABR to an acoustic click was recorded from 16 animals for indexing the TCHL. The ABR thresholds in the TCHL group (*n* = 8) were 38.06 ± 3.32 dB higher than those in the control group (*n* = 8) immediately after the eardrum perforation (P14), 14.38 ± 2.90 dB higher 14 days after the eardrum perforation (P28), and only 4.38 ± 1.55 dB higher 28 days after the eardrum perforation (P42) (Figure 1c,d). These results indicate that eardrum perforation caused conductive hearing loss immediately after the surgery, while the hearing loss could gradually recover, which is consistent with previous report (Bigelow, Kay, & Saunders, 1998).

### 3.2 Open‐field test

**Figure 2:**
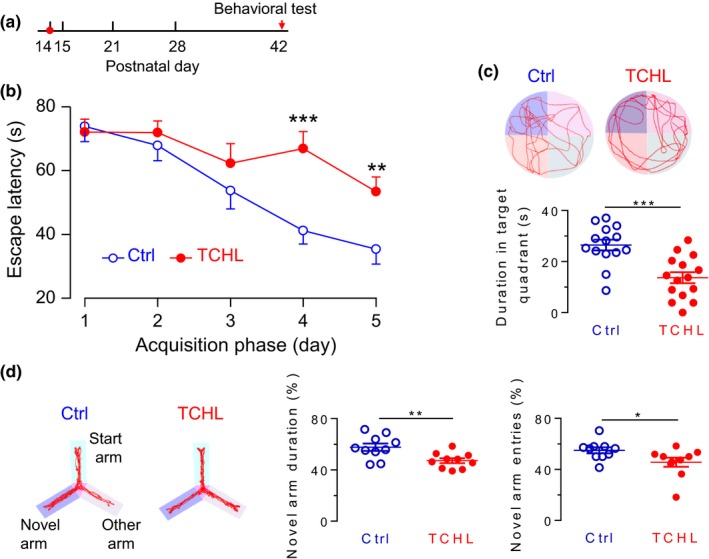
Poorer behavioral performance of the Morris water maze and the Y‐maze tests in the TCHL group than in the control group. (a) The data were collected when animals were in adulthood (P42). (b) Group data for the parameter latency to find escape platform during training trial in the Morris water maze test. (c) Group data for the duration in the target quadrant during probe trial in the Morris water maze test. Sample swim traces during acquisition phase are shown in upper panels and the target platform is indicated in blue. (d) Group data for novel arm entries and novel arm duration in the Y‐maze test. Sample activity traces are shown in left panels. For the Morris water maze test, data were collected from 14 rats in the control group and 15 rats in the TCHL group. For the Y‐maze test, data were collected from 10 rats in the control group and 10 rats in the TCHL group.***p < 0.001, **p < 0.01, *p < 0.05

To evaluate the motor ability following the TCHL, we performed open‐field test in 15 rats 4 weeks postsurgery of the eardrum perforation (P42) (Figure 2a) (the TCHL group) and in 14 rats serving as control (the control group). In the open‐field test, the total movement distance did not differ significantly between the TCHL group and the control group (3 505.01 ± 120.34 vs. 3 445.41 ± 215.54 cm, *p *>* *0.05). The sample traces of movement in the two groups are shown in Supporting Information Figure S1a. The number of total squares crossed in the TCHL group was not significantly different from that in the control group (86.93 ± 4.78 vs. 93.50 ± 4.33, *p *>* *0.05)(Supporting Information Figure S1b). The number of rearing in the TCHL group was not significantly different from that in the control group either (12.07 ± 1.11 vs. 14.71 ± 1.23, *p *>* *0.05) (Supporting Information Figure S1b).

### 3.3 Poorer scores of the Morris water maze in the TCHL group than those in the control group

To evaluate spatial memory in the rats following the TCHL, we performed the Morris water maze test in the same group of animals that were used for open‐field test. During the acquisition phase of the Morris water maze test, the escape latencies were not significantly different between two groups on Days 1, 2, or 3 (Figure 2b). However, rats in the TCHL group (*n* = 15) required a longer time period in order to find the submerged platform compared with the control group (*n* = 14) on Day 4 (66.87 ± 5.39 vs. 41.20 ± 4.21 s, *p *<* *0.001) and on Day 5 (53.43 ± 4.59 vs. 35.37 ± 4.67 s, *p *<* *0.01) (Figure 2b). On the probe trial, the TCHL group demonstrated a worse learned bias to navigate toward the target quadrant which previously contained the platform (Figure 2c). They spent less time in this quadrant than the control group did (13.65 ± 2.14 vs. 26.44 ± 2.09 s, *p *<* *0.001) (Figure 2c). Between the TCHL group and the control group, there was no significant difference in the total swim distance (1 435.56 ± 61.69 vs.1 555.94 ± 46.60 cm, *p *>* *0.05) or in mean swim velocity (18.20 ± 0.84 vs. 19.49 ± 0.72 cm/s, *p *>* *0.05) (Supporting Information Figure S1c). The results of the Morris water maze test indicate impaired spatial memory of rats in the TCHL group.

### 3.4 Poorer scores of Y‐maze tests in the TCHL group than those in the control group

We performed the Y‐maze test in a group of animals with TCHL (*n* = 10) and a group of controls (*n* = 10). The TCHL group spent significantly less time in the novel arm than the control group did (45.57 ± 3.56 vs. 54.92 ± 2.37 s, *p *<* *0.05) (Figure 2d). The TCHL group exhibited decreased number of entries in the novel arm than the control group did (47.18 ± 1.9% vs. 57.70 ± 2.89%, *p *<* *0.01) (Figure 2d). Between the TCHL group and the control group, there was no significant difference in the total distance moved (1 667.00 ± 77.92 vs.1 650.00 ± 47.76 cm, *p *>* *0.05) or in the total number of entries (40.90 ± 4.08% vs. 43.40 ± 3.48%, *p *>* *0.05) (Supporting Information Figure S1d). The results indicate that early age hearing loss in the TCHL group disrupts working memory in the Y‐maze test.

### 3.5 Impaired field potentials of the hippocampus in the TCHL group

**Figure 3:**
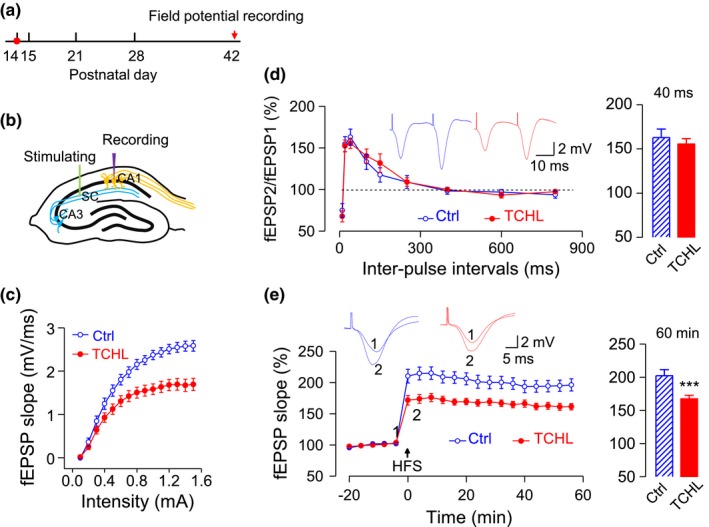
Impaired functions of the hippocampus in the TCHL group as indicated by field potentials. (a) The data were collected when animals had reached adulthood (P42). (b) Field potentials were recorded from the CA1 area with the Schaffer fiber was electrically stimulated. (c) Input–output functions of fEPSP slopes in the TCHL group and the control group. (d) PPR (fEPSP2/fEPSP1) at varying interpulse intervals (left panel) and statistical analysis for PPR at 40 ms of the interpulse interval. (e) The time course (left panel) of fEPSP slopes following HFS and the statistical analysis (right panel) for the fEPSP slopes at 60 min after HFS. The arrow indicates onset of HFS. Sample waveforms of fEPSPs prior to and following HFS are shown. Data were collected from 19 rats in the control group and 19 rats in the TCHL group. ***p < 0.001. fEPSP, field excitatory postsynaptic potential; HFS, high‐frequency stimulation; PPR, paired‐pulse ratio

We recorded field potentials of the hippocampus in a group of animals with TCHL (*n* = 19) and a group of animals as control (*n* = 19). Four weeks after the surgery for eardrum perforation (P42) (Figure 3a), the field potential evoked by stimulating the Schaffer fibers was recorded from the CA1 area of the hippocampus (Figure 3b). The fEPSP slope derived from the field potential waveform monotonously increased as the stimulus intensity increased (Figure 3c). The input/output function of the fEPSP slope in the TCHL group was significantly depressed compared with that in the control group (Figure 3c) for stimulus intensity ≥0.5 mA. There was no significant difference in PPR of the fEPSP slopes between the two groups (Figure 3d). These results indicate that TCHL impairs the basic synaptic transmission in the hippocampal CA1 area presumably through a postsynaptic mechanism.

To assess the LTP, we recorded the fEPSP slopes and normalized it to the 20 min baseline prior to HFS. The normalized fEPSP slope was counted for 60 min following HFS (Figure 3e) as the strength of LTP. Less enhancement of fEPSP slopes was observed in the TCHL group than in the control group following HFS (167.50 ± 5.24% vs. 202.36 ± 9.10%, *p *<* *0.001)(Figure 3e), indicating impaired neural plasticity of the hippocampus in the TCHL group.

### 3.6 Decreased NMDA receptor‐mediated currents in the TCHL group

**Figure 4:**
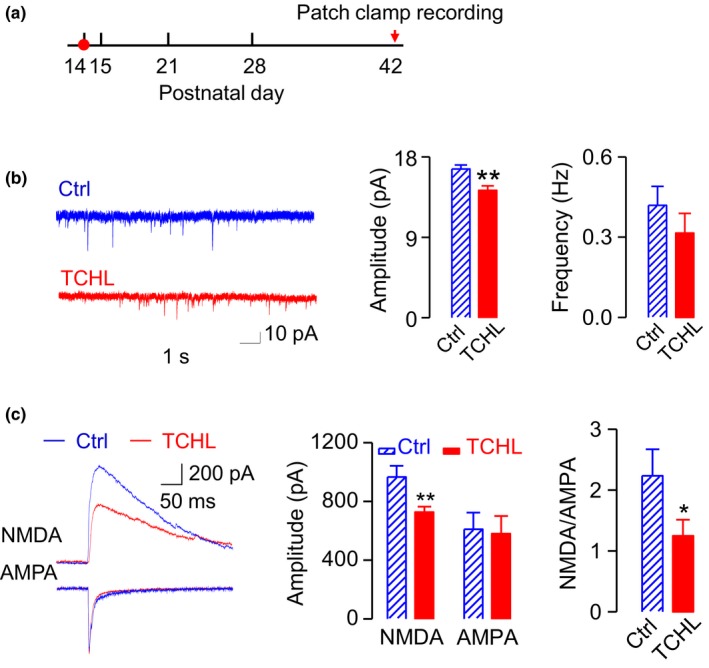
Altered NMDA receptor‐mediated mEPSCs and evoked EPSCs in the TCHL group. (a) Time schedule for data collection. (b) Sample traces of (left panels) and group data for (right panels) the amplitude and frequency of NMDAR‐mediated mEPSCs (Control: *n* = 12, TCHL: *n* = 12). (c) Sample waveforms (left panel) of and group data for amplitude (middle panel) of NMDA (Control: *n* = 19, TCHL: *n* = 21) and AMPA (Control: *n* = 13, TCHL: *n* = 13) currents evoked by stimulating the Schaffer fibers, and group data for ratio of NMDAR‐mediated evoked EPSCs to AMPA receptor‐mediated evoked EPSCs (NMDAR/AMPAR ratio) (Control: *n* = 8, TCHL: *n* = 8). *p < 0.05, **p < 0.01. NMDAR: NMDA receptor; mEPSC: miniature excitatory postsynaptic current

Whole‐cell patch‐clamp recordings were made from neurons in the CA1 area of hippocampal slices in animals 4 weeks after the surgery for eardrum perforation (P42) (Figure 4a) and from those in animals as control (total 96 neurons in 39 rats, Table 1). There was no significant difference in the resting membrane potential (‐59.37 ± 1.53 vs. ‐59.20 ± 2.27 mV, *p *>* *0.05) or in the input resistance (307.35 ± 56.73 vs. 291.55 ± 51.92 MΩ, *p *>* *0.05) between the TCHL group (*n* = 8) and the control group (*n* = 9) (Supporting Information Figure S2). However, the amplitude of NMDA receptor‐mediated mEPSCs was significantly reduced (14.27 ± 0.51 pA vs. 16.64 ± 0.45 pA, *p *<* *0.01) in the TCHL group (*n* = 12) compared with those in the control group (*n* = 12), while the frequency did not differ significantly (0.32 ± 0.07 vs. 0.42 ± 0.07 Hz, *p *>* *0.05) (Figure 4b).

EPSCs were evoked and recorded with the stimulating electrode placed in the Schaffer collateral. The amplitude of the NMDA receptor‐mediated EPSCs was decreased significantly (727.50 ± 37.89 vs. 965.55 ± 76.17 pA, *p *<* *0.01) in the TCHL group (*n* = 21) than in the control group (*n* = 19) (Figure 4c), while that of the AMPA receptor‐mediated EPSCs did not differ significantly (579.79 ± 121.26 vs. 611.48 ± 112.53 pA, *p *>* *0.05) between the TCHL group (*n* = 13) and the control group (*n* = 13) (Figure 4c). The NMDA/AMPA ratio was significantly decreased (1.25 ± 0.26 vs. 2.24 ± 0.43, *p *<* *0.05) in the TCHL group (*n* = 8) than in the control group (*n* = 8) (Figure 4c).

### 3.7 NR2A receptors were upregulated, but NR2B receptors downregulated in the TCHL group

**Figure 5:**
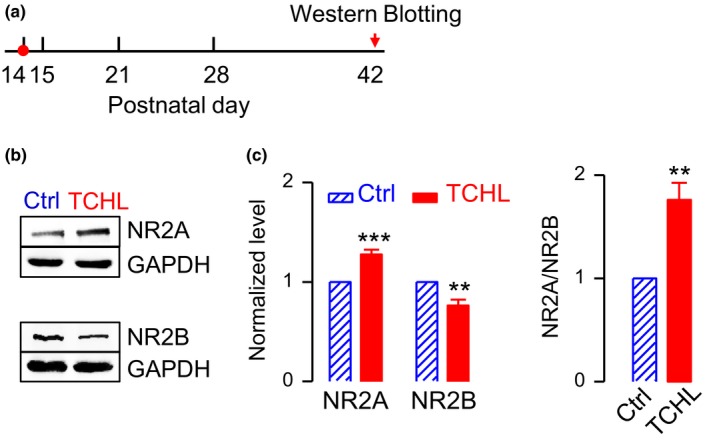
Expression level of NR2A and NR2B receptors. (a) Time schedule for data collection. (b) Western blot analysis of NR2A and NR2B. (c) Group data for normalized expression level of NR2A and NR2B receptors (left panel) and for NR2A/NR2B ratio (right panel). Data were collected from nine rats in the control group and nine rats in the TCHL group. ***p < 0.001, **p < 0.01

We tested NR2A and NR2B content by western blot analysis in nine rats 4 weeks after the surgery for eardrum perforation (P42) (Figure 5a) and nine rats as control (Table 1). Figure 5 shows the sample bands of western blot. The level of NR2A was significantly increased (1.28 ± 0.046 vs. 1.00 ± 0.00, *p *<* *0.001) in the TCHL group (*n* = 9) in comparison with that in the control group (*n* = 9), whereas that of NR2B significantly decreased (0.76 ± 0.058 vs. 1.00 ± 0.00, *p *<* *0.01) (Figure 5c). The ratio of NR2A/NR2B was significantly increased in the TCHL group in comparison with that in the control group (1.76 ± 0.16 vs. 1.00 ± 0.00, *p *<* *0.001) (Figure 5c).

### 3.8 Decreased dendritic spines and PSD in the TCHL group

**Figure 6:**
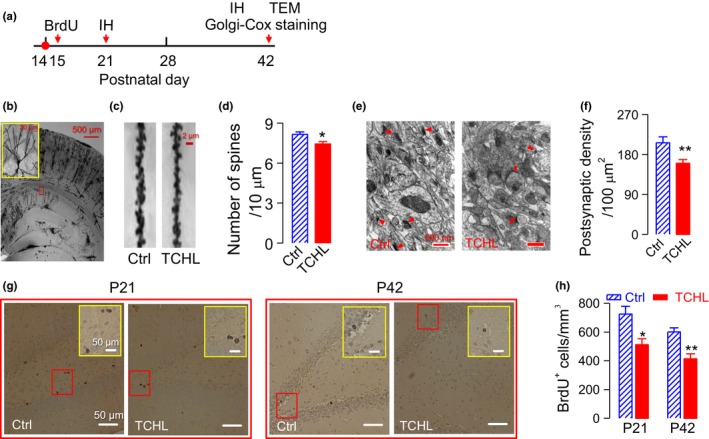
Dendritic spines, postsynaptic density, and BrdU^+^ cells in the hippocampus. (a) Time schedules for data collection. (b) A photomicrograph of a representative Golgi‐Cox‐impregnated brain slice (100 μm). The upper left panel indicates the enlarged area from the red solid box. (c) Representative dendritic spines from CA1 pyramidal neurons. (d) Group data for count of dendritic spines of pyramidal cells (Control: *n* = 43 neurons in six rats, TCHL: *n* = 48 neurons in six rats). (e) Sample TEM showing the postsynaptic density (red arrows). (f) Group data for postsynaptic densities (Control: *n* = 8, TCHL: *n* = 10). (g) Sample BrdU‐labeled cells in the hippocampus. The upper right panel indicates the enlarged area from the red solid box. (h) Group data for BrdU^+^ cells (Control: *n* = 6, TCHL: *n* = 6). **p < 0.01. *p < 0.05. IH: Immunohistochemistry; TEM: transmission electron micrograph

Dendritic spines and PSD of hippocampal neurons were evaluated 4 weeks after the surgery for eardrum perforation (P42) using Golgi‐Cox staining (Figure 6a). Figure 6b shows a representative CA1 pyramidal neuron in a Golgi‐Cox‐impregnated brain slice. The neurons in the TCHL group (*n* = 48 neurons in 6 rats) had significantly less dendritic spines than the control group did (*n* = 43 neurons in 6 rats) (7.43 ± 0.17/10 μm vs. 8.16 ± 0.17/10 μm, *p *<* *0.05) (Figure 6c,d). PSD was observed by TEM as an electron‐dense region at the membrane of a dendritic spine (Figure 6e). The PSD in the TCHL group (*n* = 10) was significantly lower than that in the control group (*n* = 8) (160.00 ± 8.50 vs. 206.25 ± 13.15, *p *<* *0.01) (Figure 6f).

### 3.9 BrdU‐labeled neurons were decreased in the TCHL group

Neurogenesis would be decreased in case of impaired learning and memory (Bruel‐Jungerman, Rampon, & Laroche, 2007; Deng, Aimone, & Gage, 2010). We thus calculated the number of newborn cells in the hippocampus of six rats that experienced a TCHL and six rats that served as control. BrdU was injected at the second day following the bilateral eardrum perforation, and immunohistochemistry was performed at 7 days (P21) and 4 weeks (P42) following the eardrum surgery (Figure 6a). The BrdU‐labeled cells were significantly less at 7 days (511.57 ± 42.05 vs. 724.72 ± 54.07, *p *<* *0.05) and at 4 weeks (414.53 ± 33.53 vs. 601.07 ± 27.30, *p *<* *0.01) following the eardrum surgery in the TCHL group (*n* = 6) than those in the control group (*n* = 6) (Figure 6g,h). The results indicate decreased neurogenesis in rats having experienced a TCHL.

### 3.10 CTB tracing of neural projections from the auditory cortex to the hippocampus

**Figure 7:**
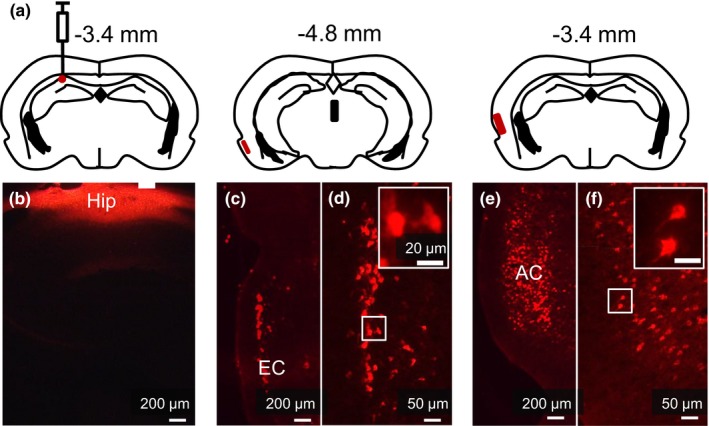
Retrograde tracing experiment showing neural projections from the auditory cortex to the hippocampus. (a) 2% CTB conjugated with Alexa Fluor 594 was injected into the CA1 area. (b) The hippocampus exhibited intense staining in red in the left region. (c–d) Higher magnification of the entorhinal cortex showing the signals. (e–f) The signals were present in the auditory cortex. AC: auditory cortex; CTB: cholera toxin subunit B; EC: entorhinal cortex; Hip: hippocampus

In order to determine whether there are neural projections from the auditory pathway to the hippocampus, we injected CTB in the CA1 area of the left hippocampus with the injection tip lowered to 2.2 mm of the ventral region in one rat (Figure 7a,b). The putative location of the hippocampal injection site was confirmed by observation of the retrograde labeling in the entorhinal cortex (Figure 7c,d). The fluorescence signals were shown in the auditory cortex (Figure 7e,f), indicating neural projections from the auditory cortex to the hippocampus.

## 4 DISCUSSION

In the present study, we induced a TCHL in young rats at the critical period of auditory development (P14) by bilateral eardrum perforation. The TCHL impairs spatial memory of the rats in adulthood, which is evidenced by the poorer scores for the Morris water maze and the Y‐maze tests in the TCHL group than those in the control group (Figure 2). The memory impairment may well stem from the abnormal development of the hippocampus in rats with a TCHL, which is evidenced by the decreased input/output functions of the field potentials (Figure 3c,d), the depressed LTP (Figure 3e), the altered expression level of NMDA receptors (Figures 4 and 5), the decrease in number of reduced synaptic spines (Figure 6b–d), the reduced postsynaptic density (Figure 6e,f), and the reduced BrdU‐labeled neurons (Figure 6g,h) in the hippocampus of the TCHL group. Our findings clearly indicate that negative impacts of auditory sensory deprivation at early life actually span beyond the auditory system to the hippocampus. In other words, our study shows that normal auditory inputs are a prerequisite for normal development of memory functions.

The abnormal development of the hippocampus was at least partially responsible for the memory impairment in the TCHL group. The hippocampus is an essential brain structure for episodic memory (Gaffan, 1994; Olton & Samuelson, 1976; Scoville & Milner, 1957; Steele & Morris, 1999) and spatial memory (Morris, Garrud, Rawlins, & O’Keefe, 1982; Muller, Stead, & Pach, 1996). In the TCHL group, a decrease in magnitude of input/output functions of the field potential (Figure 3c), a decrease in number of neuronal spines (Figure 6b–d), and a decrease in PSD (Figure 6e,f) indicated that the basic synaptic transmission in the hippocampus was impaired. The depressed LTP (Figure 3d) and the abnormal expression of NMDA receptors (Figure 5) indicate impaired neural plasticity of the hippocampus. In addition, a decrease in BrdU‐labeled neurons (Figure 6g,h) indicates lower level of neurogenesis in the hippocampus, while neurogenesis of the hippocampus in both rodents and humans is considered to be significant for maintenance of the memory function throughout life (Eriksson et al., 1998; Kuhn, Dickinson‐Anson, & Gage, 1996). These abnormalities in the hippocampus of the TCHL group due to early auditory deprivation surely contribute to the impairment of spatial memory.

The impaired behavioral performance for the Morris water maze and the Y‐maze in the TCHL group (Figure 2) was not likely contributed by the impaired auditory functions. Most previous studies regarding consequences of early auditory deprivation focus on structural and functional changes in the central auditory systems (Polley et al., 2013; Popescu & Polley, 2010; Xu et al., 2007), or on the auditory cognitive performance. These studies show that animals with a hearing loss at young age will undergo abnormal auditory development and have impaired auditory functions. In the present study, the impaired hearing sensitivity following bilateral eardrum perforation did recover, although did not completely recover (Figure 1d), presumably due to the scars on the eardrum (Figure 1b). In addition, we assume that the central auditory processing, including auditory cognitive processing, in the TCHL group is also impaired. However, the auditory deficits should not interfere with the tests of the Morris water maze and the Y‐maze because these tests do not involve any auditory tasks.

Anatomical and functional connections between the auditory system and the hippocampus may form a neural basis for the dependence of hippocampal development on normal auditory inputs. In the present study, we show that there are direct neural projections from the auditory cortex to the hippocampus as indicated by the CTB tracing experiment (Figure 6). There are quite a few studies to show responses of the hippocampus to auditory signals (Itskov, Vinnik, Honey, Schnupp, & Diamond, 2012; Miller & Freedman, 1995) and to show that adverse disturbances, such as acoustic trauma to the auditory systems (Goble, Moller, & Thompson, 2009; Liu et al., 2016), will alter functions of the hippocampus, which indicates its functional connections to the auditory system. Because of these close anatomical and functional connections, one can imagine that the developmental maturation of the hippocampus accompanies bombardment of auditory information, and deprivation of auditory inputs will derail the normal course of hippocampal development as demonstrated by the present study.

Our study has a clinical implication in otitis media or middle ear infection, in young children. More than half of children will experience at least one episode of otitis media by their first birthday (Teele, Klein, & Rosner, 1989), which results in a conductive hearing loss and consequently auditory sensory deprivation. Children with a history of middle ear infection seem to have poorer learning and cognitive capacities than their peers do (Reichman & Healey, 1983; Williams & Jacobs, 2009). However, it is unclear whether the auditory sensory deprivation of those children in early life is causally related to their impaired learning and cognitive functions because child development appears to depend on the interrelationship between many factors, such as gender, birth status, genetic predisposition, and socioeconomic status. Using a rat model, the present study has shown that a TCHL in early life can impair spatial memory in adulthood. Our study provides a line of evidence from animal research perspective to support that a negative consequence of otitis media in human infants may span beyond the auditory system to the high brain functions.

## CONFLICT OF INTEREST

None declared.
