# Supplementary material for: Temporary conductive hearing loss in early life impairs spatial memory of rats in adulthood
Source: Brain Behav. 2018 May 31;8(7):e01004. doi: 10.1002/brb3.1004 (PMC6043706; doi:10.1002/brb3.1004)
Supplement: Supplementary file 1 [file BRB3-8-e01004-s001.pdf]

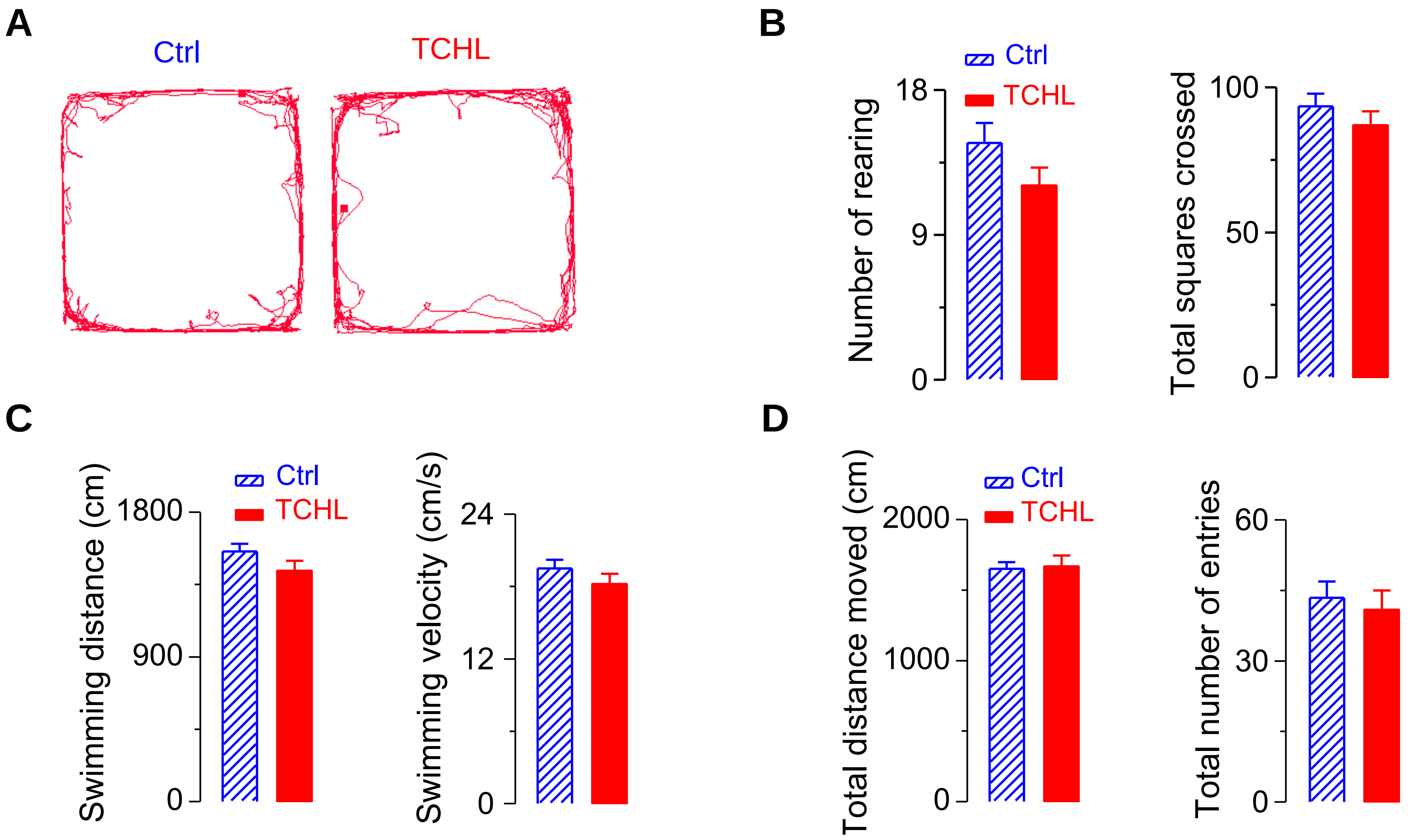

Supplementary Figure 1. Behavioral outcomes showing motor functions of the rats in the TCHL group (n = 15) and in the control group (n = 14). (A) The sample traces during acquisition phase in open field test. (B) Total squares crossed and number of rearing in open field test. (C) Swimming distance and swimming velocity in Morris water maze task. (D) Total distance moved and total number of entries in Y maze task.
