# Supplementary material for: Temporary conductive hearing loss in early life impairs spatial memory of rats in adulthood
Source: Brain Behav. 2018 May 31;8(7):e01004. doi: 10.1002/brb3.1004 (PMC6043706; doi:10.1002/brb3.1004)
Supplement: Supplementary file 2 [file BRB3-8-e01004-s002.pdf]

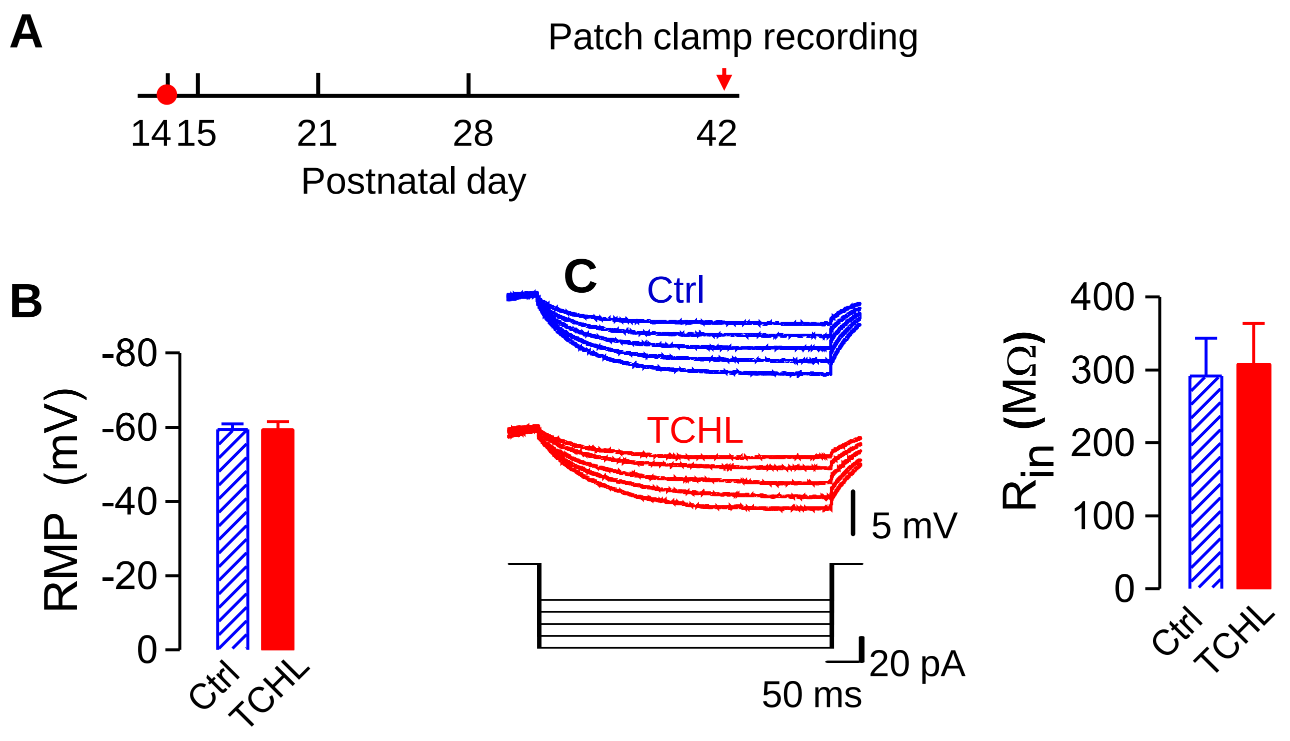

Supplementary Figure 2 The intrinsic properties of neuronal membrane in the CA1 area of the hippocampus in the TCHL group and the control group. (A) Schedule for data collection. (B) Group data for the resting membrane potential (BMP). (C) Sample traces of membrane potentials measured with injection of hyperpolarizing step currents (left panel) and group data for the derived input resistance (right panel) (TCHL: n = 8, Control: n = 9).
